# Interactively AUDIT Your Growth Curves with a Suite of R Packages

**DOI:** 10.1534/g3.119.400898

**Published:** 2020-01-23

**Authors:** Nicolas P. J. Coutin, Guri Giaever, Corey Nislow

**Affiliations:** * Department of Biochemistry and Molecular Biology, Faculty of Medicine, and; † Faculty of Pharmaceutical Sciences, University of British Columbia, Vancouver, B.C., Canada, V6T 1Z3

**Keywords:** growth curve, chemical genetics, yeast, fitness

## Abstract

Bottlenecks often occur during data analysis when studying microbial growth in liquid culture at large scale. A researcher can collect thousands of growth curves, repeated measures of a microbial liquid culture, at once in multiple micro titer plates by purpose-built robotic instruments. However, it can be difficult and time-consuming to inspect and analyze these data. This is especially true for researchers without programming experience. To enable this researcher, we created and describe an interactive application: Automated Usher for Data Inspection and Tidying (AUDIT). It allows the user to analyze growth curve data generated from one or more runs each with one or more micro titer plates alongside their experimental design. AUDIT covers input, pre-processing, summarizing, visual exploration and output. Compared to previously available tools AUDIT handles more data, provides live previews and is built from individually re-usable pieces distributed as R packages.

The study of microbial growth in liquid culture is now applied at large scale for diverse applications. The quantitative study of the growth of microbial cultures dates back more than 70 years to the work of Monod and others (Monod 1949; Koch 1970). Repeated measures of the density of cells in a suspension over time yield a growth curve. Researchers summarize these curves to a small set of parameters. These values are then compared to yield the relative fitness of the microbial system when probed. Robotic manipulation of Micro Titer Plates MTPs in purpose-built instruments has allowed studying thousands of conditions in parallel. For example, Lee *et al.* (2014) validated hits from a genomic approach in large scale drug discovery. Others performed High Throughput Screening HTS directly to study drug interactions and the saline response in yeast (Cokol *et al.* 2011; Warringer and Blomberg 2003; Warringer *et al.* 2003).

A limiting step in HTS is often data analysis. Specifically, manipulating, inspecting and tidying growth curve data across multiple MTPs and multiple runs can be tedious or impossible. Further, different preprocessing and summarization methods are appropriate to apply depending on the experiment and instrument setup (Malo *et al.* 2006; Blomberg 2011; Stevenson *et al.* 2016). Custom requirements complicate data analysis in HTS. They also suggest that automated software pipelines may not be appropriate for all applications. The custom tooling already available for growth curve analysis highlights this fact (Table S1).

We describe an Automated Usher for Data Inspection and Tidying (AUDIT) for growth curves. The AUDIT application allows the user to analyze growth curve data, joined with their experimental design, for one or more MTPs across one or more runs. AUDIT guides the user through data input, preprocessing, summarization, visual exploration and output. AUDIT automatically tidies raw experimental data in different formats and joins experimental design data. This interactive web application runs locally or over the web. We wrote it in the shiny R and Javascript (JS) framework (Chang *et al.* 2019). This application’s code relies on independently written modules and is open-source. AUDIT’s design enables modification and re-use.

To demonstrate AUDIT, we study the effect of two chemical probes on the growth of *Saccharomyces cerevisiae* reference and gene deletion strains. The complete source code for AUDIT, along with example data, is freely distributed as an R package for easy installation and is available online at https://github.com/npjc/audit.

## Materials and Methods

### Reading and tidying raw input

AUDIT considers two types of input files. Measures files contain the raw repeated measures data, usually produced directly by the growth instrument’s built-in software. The measure is the property being measured about each well (*e.g.*, Optical Density (OD), Absorbance at 620 nanoMeters ($A_{620nm}$)) at repeated time intervals. Design files contain other information that may vary across wells and makes up the experiment design (*e.g.*, strain, drug, media). These files are usually produced by the researcher. These files are usually produced manually in a spreadsheet application (*e.g.*, plater format (Hughes 2016)).

AUDIT will accept input files in six formats. Raw measures data can be in Yeast Grower (YG) format (Proctor *et al.* 2011), Bioscreen C format (Oy Growth Curve), Corey Guri Twelve (CG-12) (S and P Robotics) format and Genome Processor One (GP1) (S and P Robotics) format. For a description of the CG-12 and GP1 high throughput microbial growth instruments see Figure S1. Design files can be in plater format (Hughes 2016) or in generic comma-separated values Comma Separated Values (CSV) format. File formats are automatically detected for each file using the regular expressions in Table S2. For details on how we defined file formats, see Defining file formats in Supplementary Materials.

AUDIT reports runtime in seconds, minutes or hours based on user input. Some instruments report absolute time in addition to or instead of runtime (elapsed time). Where appropriate, reading modules can report datetime in ISO8601 format. Similarly, parsers can output the optional measure_type column to differentiate more than one measurement value taken at the same runtime interval.

#### Multiple files:

If multiple files are uploaded, AUDIT will join measures and design files together. To do this it applies a heuristic based on file type and number. If given two files, if one is a design file, join onto the measures file and create a single run. If given more than two files, and only one design file, join onto each of the measures files and create one run per measures file. If given more than two files and there are multiple design files, join measures and designs by partial name matching (*e.g.*, run1_design.csv would match to measures file run1.txt).

#### Built-in example data:

AUDIT provides example data files in each format and for the case study described herein. Users can load these files by clicking the links in input panel. AUDIT parses example data files identically to user uploaded content. They are also distributed as part of AUDIT’s source repository in the example-data directory (see Data Availability).

### Preprocessing Measures

Preprocessing options in AUDIT allow for log transformation, smoothing, background correction and calibration. First, data can be log-transformed and then smoothed sequentially with a running median and mean filter. Median smoothing reduces high amplitude noise at single time points and mean smoothing reduces low amplitude high frequency noise (Fernandez-Ricaud *et al.* 2016). AUDIT can transform curves so that they always increase. This transformation is to reduce bias introduced by bubble formation, cell aggregation or drug precipitation (Fernandez-Ricaud *et al.* 2016). Lastly, because OD measurements increase non-linearly with cell densities (Stevenson *et al.* 2016; Fernandez-Ricaud *et al.* 2016; Blomberg 2011), an arbitrary calibration function can be applied.

#### Log transformation and smoothing:

Sigmoid growth models assume that the measured signal has been log-ratio transformed according to: $log\left(N/N_{0}\right)$, where N is the current number of organisms and $N_{0}$ is the initial number of organisms (Zwietering *et al.* 1990). To enable this kind of transformation generally, raw measures (y) may be log-ratio transformed: log(y/min(y)) . We use the minimum measure (min(y)) instead of the first measure (y[1]) in case of noisy initial measurements. AUDIT can smooth growth curves with a running median filter followed by running mean smoothing (Fernandez-Ricaud *et al.* 2016). The width of each filter can be any odd integer less than the length of the input values. Finally, it is possible to enforce that the transformed measures be increasing. Here, the software tests if each measure is equal or larger than the previous value. If this is not the case, the current measure is replaced with the previous value against which it was compared.

#### Background correction and calibration:

Background correction and calibration in AUDIT use functional interfaces. The user inputs a formula into each text box and AUDIT applies it each well. The variable y represents the incoming values, therefore a calibration function y would output the incoming values unchanged. This flexible interface makes it possible to interactively execute a wide variety of methods.

To increase the specificity of the measured value to the intended signal the user can perform background subtraction. For example, to subtract a fixed background value: y−0.081. Dynamic background subtraction is also possible. Specifying y−min(y) will remove the minimum measure from each well. Alternatively, y−mean(y[1:5] will remove the mean of the first 5 values.

To correct for a non-linear relationship between the observed (measured) signal and the actual (true) signal, the user can provide a calibration function. Users can perform a calibration to relate the absorbance or OD measured by the spectrophotometer directly to the cell number or dry weight (Pringle and Mor 1975). Table S3 provides examples of how to input a calibration function in AUDIT. We recommend empirically determining a calibration function for each experimental setup. Figure S2 gives an example. For more details see Calibration procedures in Supplementary Materials and Stevenson *et al.* (2016).

### Summarizing Growth Curves

#### Smooth spline interpolation and self-starting model fits:

After preprocessing, the user chooses one of five methods to estimate summary metrics. These are smooth spline (R Core Team 2019), Gompertz (Zwietering *et al.* 1990), logistic (Zwietering *et al.* 1990), Richards (Zwietering *et al.* 1990) and manual (see Arbitrary model specification via manual mode). AUDIT fits each curve via non-linear (weighted) least-squares estimation. For a visual comparison of these methods on the same data see Figure S4.

After fitting, the software computes general summary statistics for each curve (Table S4, Figure S3). It estimates the maximum measure value reached during the assay and the time at which it (first) occurs: $max{\left(y\right)}_{x,y}$ . From the dy/dt curve, we take the maximum to yield the maximum rate: $max{\left(dy\right)}_{m}$ . Similarly, $max{\left(dy\right)}_{x,y}$ is the point on the growth curve where the slope is at its (first) global maximum. We also estimate the x- and y-intercepts for the maximum slope line: $max{\left(dy\right)}_{b}$, $max{\left(dy\right)}_{x_{y=0}}$ . Last, we compute the area under the curve by integrating over the range of the curve fit with respect to time: $\int_{x=0}^{x=t_{max}}f\left(x\right)$ . We refer to this value as fit_int in AUDIT.

The application will also estimate model specific coefficients, via non-linear least-squares, for each curve. For example, metrics for the built-in Gompertz, logistic, and Richards methods: the asymptotic growth limit (A), the maximum growth rate (μ) and the lag time (λ). In an alternative implementation, Kahm *et al.* (2010) compared the performance of model and model-free methods on example data. They concluded that a spline fit produced more accurate estimates of the characteristic growth parameters and that model fits may lead to unreliable predictions. For the original derivation of A, μ and λ see Zwietering *et al.* (1990).

The growr module that implements these methods provides additional functions not available interactively. It is possible to define and estimate additional user-defined metrics at the R console. For example, we implemented an additional metric: the minimum doubling time (Figure S5). See Extending AUDIT with new metrics in Supplementary Materials for details.

#### Arbitrary model specification via manual mode:

AUDIT provides an extensible model interface that makes it possible to interactively specify and fit arbitrary models. To engage this mode the user selects the ‘manual’ estimation method in the summarize panel. They can then specify any model that is expressible as an R formula. Specifically, taking the form ‘y ?x’, where y is the preprocessed measures data and x is the runtime. AUDIT generates model fits and estimates coefficients as above, but it requires a user-supplied initial estimate of each model parameter. To do this, the user supplies starting values as named comma-separated values in the form: ‘a1 = 0.05,...’. As an example, Figure S6 shows the output of manually specifying the Brody growth model ($y\left(t\right)=\alpha -\left(\alpha -w_{0}\right)\text{*}exp\left(-k*t\right)$) (Brody 1966). The formula for this model is ‘y ?a - (a - w0) * exp(-k * x)’ and the starting estimates chosen are ‘a = 1,w0 = 0.5, k = 0.00003’. See Extending AUDIT with new models in Supplementary Materials for implementation details.

#### Curve quality and model goodness of fit:

To summarize the quality of curve fits AUDIT computes common model statistics. R’s built-in smooth.spline() and nls() methods provide these quality metrics. In the case of cubic (smooth) splines: the smoothing parameter (spar), the equivalent degrees of freedom (df), the underlying criterion minimized (crit), the (weighted) residual sum of squares (penalized criterion) and cross-validation score (cv.crit). For all other models, including custom models fits: the square root of the residual variance (sigma), whether the fit successfully converged (isConv), the achieved convergence tolerance (finTol), the data’s log-likelihood under the model (logLik), the Akaike Information Criterion (AIC), the Bayesian Information Criterion (BIC), the deviance and the residual degrees of freedom (df.residual).

### Exploring Results

#### Experiment-at-a-glance:

To visualize a summary metric across an experiment, the user first chooses which metric to display with a drop-down menu in the explore panel. The software then plots each well in each plate of the experiment as a rectangle and maps data to plate or well properties. We call this an MTP view plot. AUDIT’s experiment-at-a-glance fills each well with a color representing the value of the summary metric. The viridis package provides a color scale where the user perceives equal steps in data as equal steps in color. This type of scale enables accurate visual comparison (Garnier 2018).

To export this graphic as a Portable Network Graphics (PNG) file, the user can right-click on the image and select the ‘Save Image As...’ item from the contextual menu. All other images produced by AUDIT inherit this behavior.

#### Applying reference/target groups:

To define reference and target wells, separate them with a ‘dash and greater than’ arrow (->). Each side can contain multiple sets of wells in a comma separated list. Rectangular selection is also possible by using a colon (:) to define the range of the selection. Finally, to apply the grouping to a specific plate, prefix the grouping by its name and an exclamation point (!).

Users can apply one or more groups with the shorthand syntax [plate name]![ref wells]->[target wells]. For example 1!A01->A01:H12 will make well A01 the reference for all 96 wells of a MTP named 1 with 8 rows and 12 columns. Omitting the plate name (A01->A01:H12) applies the grouping to each plate in the run. To apply multiple groups, the user specifies one per line. Groups can use wells multiple times and they are not mutually exclusive. Currently, all groups must apply to plates of the same dimensions.

After parsing the grouping shorthand, AUDIT adds two additional columns to the summarized data to encode the grouping information into tabular format. The group column contains the group name while the is_ref column indicates which wells in that group are the reference wells with TRUE or FALSE.

### Data output

AUDIT can output tidied raw and preprocessed measures data, summary metrics data, and model quality statistics. The user can download these CSV files from the output pane. The outputted measures data contains all experimental design data and/or grouping joined during the session. Outputted results are consistent for a given set of input data, preprocessing parameters and summary method.

### Data availability

AUDIT is freely available at https://nicolascoutin.shinyapps.io/audit/ and is distributed as an R package from https://github.com/npjc/audit. Step-by-step installation instructions are available in the repository’s README.md document.

AUDIT is an interactive interface to multiple, independently useful, modules. Table 1 describes and links to their freely available source code and documentation.

**Table 1:** Description and Repositories for AUDIT Modules

Name*^a^*	Description	URL
mtpview	Easily view your MTP data	https://github.com/npjc/mtpview1
readcg12	Parse raw input files from CG-12 instruments (S&P Robotics)	https://github.com/npjc/readcg12
readgp1	Parse raw input files from GP1 instruments (S&P Robotics)	https://github.com/npjc/readgp1
readyg	Parse raw input files from YG (ACCESS) files [Proctor *et al.* (2011) Proctor, Urbanus, Fung, Jaramillo, Davis, Nislow, and Giaever]	https://github.com/npjc/readyg
readbioscreen	Parse raw input files from BioScreen instruments (Oy Growth Curve)	https://github.com/npjc/readbioscreen
growr	Preprocessing, summarizing and grouping of growth curves.	https://github.com/npjc/growr

a Each module is an R package available from github with its own documentation and additional functions not directly available in AUDIT.

To make it easy to test AUDIT, we distribute example data as part of the application’s source directory: https://github.com/npjc/audit/tree/master/inst/audit-main/example-data. The user can also load these files, without downloading them first, within the application’s input panel. Output data produced from the movie and figures presented herein are also distributed with AUDIT’s source: https://github.com/npjc/audit/tree/master/inst/audit-main/example-data-output.

Additionally, the following supplementary files have been deposited at figshare https://doi.org/10.25387/g3.11691870:

- Movie S1.avi: Movie file demonstrating AUDIT session matching figures in main text.
- supplementary-materials.pdf: Contains all supplementary tables, figures, listings and text referenced but not presented in the main article.

## Results

### Overview of AUDIT

AUDIT is an interactive web application that provides a high-level interface for large-scale growth curve analysis (Movie S1). Researchers do not need programming experience to use it. AUDIT guides the user through data input, preprocessing, summarization, visual exploration and data output. We wrote AUDIT in the Shiny framework using the R and JS programming languages (Chang *et al.* 2019; R Core Team 2019). Researchers can host the application online or run it locally on their machine. Making the application available over an internal or public website makes for the easiest end-use but requires maintaining a dedicated Shiny server. In addition, an administrator needs to manage user permissions to permit uploading and downloading files from the server. Installing AUDIT locally, on the same machine where it will be used, removes the need for a network connection. AUDIT will be more responsive locally, because it will not have to wait on data crossing the network. In our testing AUDIT is fast enough to enable interactive use: with each step completing in fractions of a second.

#### Architecture and conventions:

**Figure 1:**
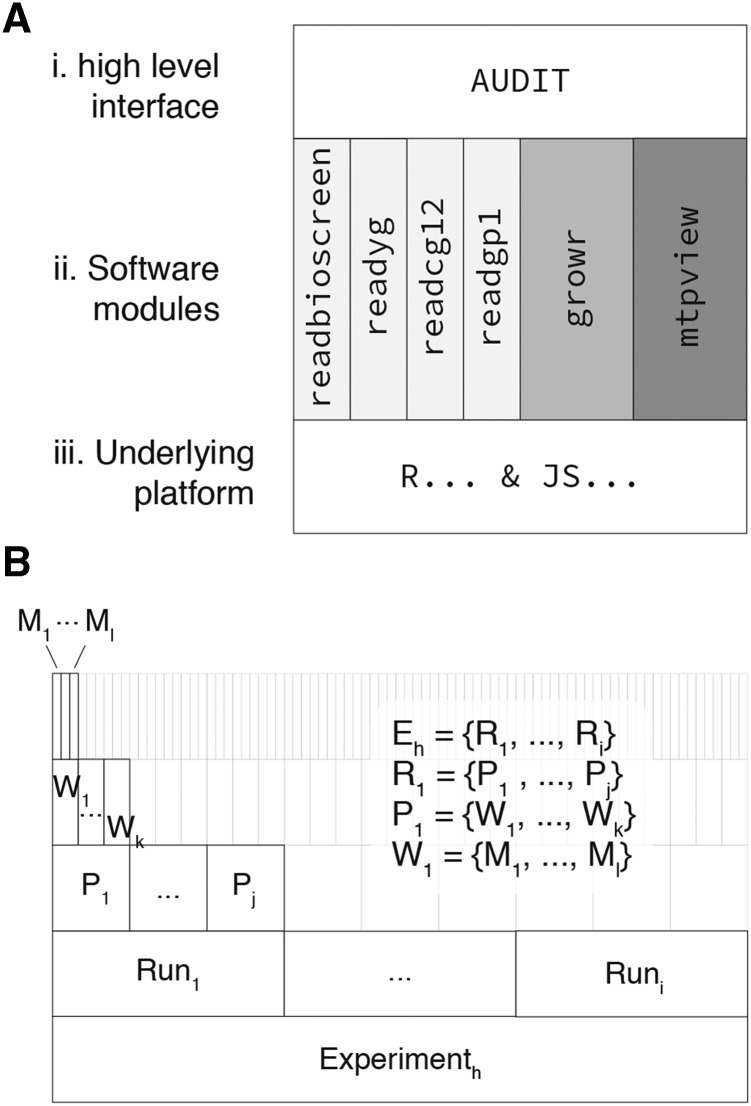
Overview Architecture and Conventions. A. AUDIT is an interactive, higher level, interface (i) atop a set of software modules/packages (ii) written and distributed via the underlying software platforms in R and JS (iii). The individual function groups (ii) are available as individual packages to enable re-use outside of the context of AUDIT. B. The stratification of individual measures across an experiment. $Experiment_{h}$ contains the set of all runs from 1 to *i* ($Run_{1},\ldots ,Run_{i}$). Each Run contains the set of all plates from 1 to *j* ($P_{1},\ldots ,P_{j}$). Each plate contains the set of all wells from 1 to *k* ($W_{1},\ldots ,W_{k}$). Each well contains the set of measures from 1 to *l* ($M_{1},\ldots ,M_{l}$).

AUDIT is a high-level interface to individual software modules (Figure 1A). This modular design makes it easy to reuse a subset or superset of the functionality provided. The software modules, individually available as R packages, each serve a different purpose (Table 1). The readbioscreen, readyg, readcg12, readgp1 implement functions that read and tidy raw repeated measures data from Bioscreen, YG (ACCESS), CG-12 and GP1 instruments, respectively. The growr package provides functions related to the preprocessing, modeling and summarization of growth curve data. Finally, the mtpview package provides functionality to visualize growth curves, summary metrics and experimental variables within one or more MTPs by extending the plotting library ggplot2 (Wickham 2016).

AUDIT defines a convention for grouping repeated measures across an experiment (Figure 1B). An experiment contains one or more runs. Each run contains one or more MTPs with its own set of wells. Each well has one or more measures. Therefore, we define each experiment as a set of observations each belonging to precisely one run, plate and well. This convention makes the manipulation and output of data consistent across a variety of input sources.

#### Input: Automatic parsing and merging of measures data with experimental design files:

**Figure 2:**
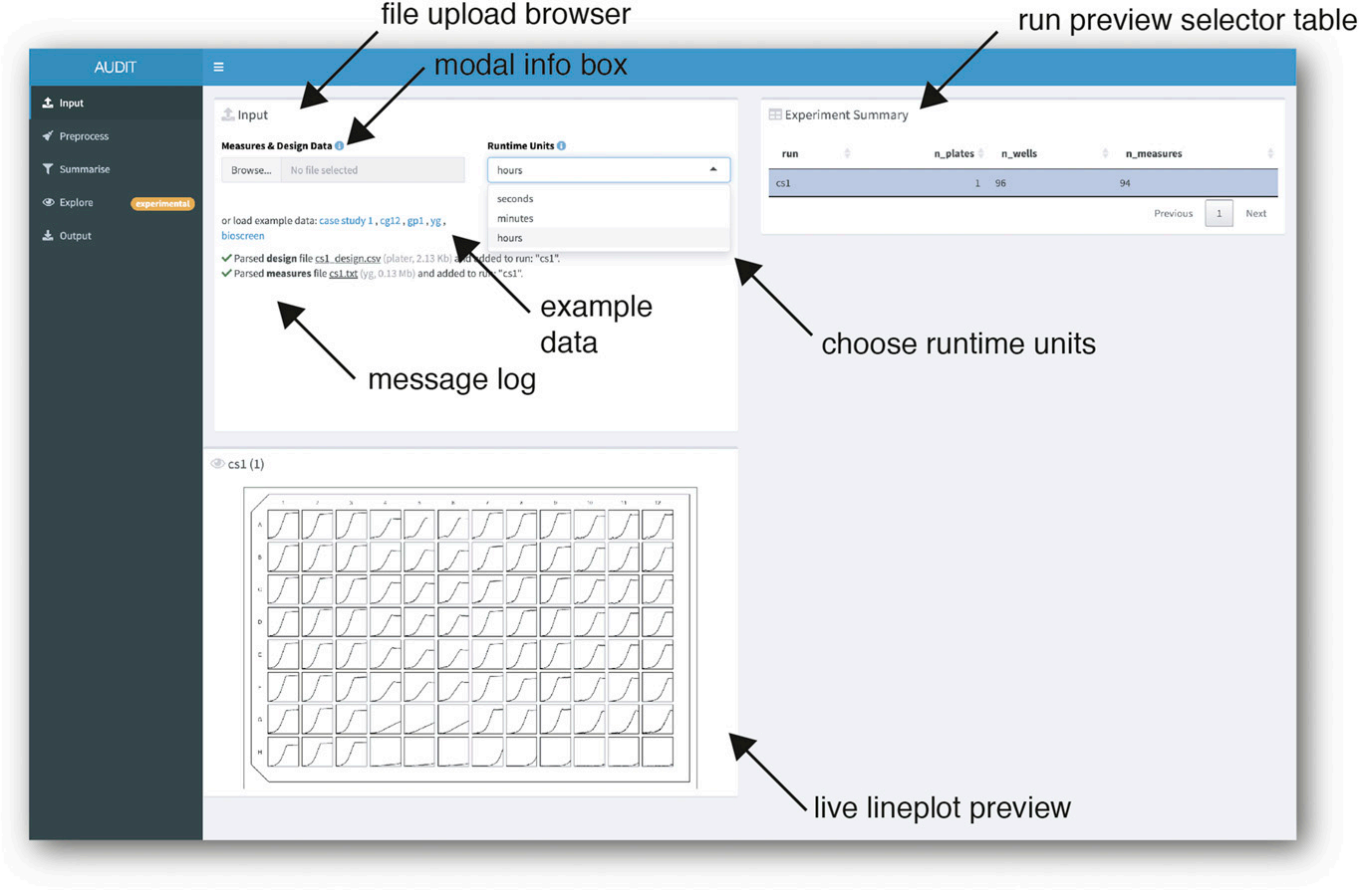
Automatic parsing and tidying of design and measures files uploaded to AUDIT. Screenshot of application’s input panel. Users upload one or more files via the file browser (arrow, top-left). Most inputs in AUDIT have a clickable ‘I’ that, when clicked, will display modal help box with additional information (arrow, second from top-left). After upload, files are automatically parsed and joined into runs. AUDIT logs parsing and joining actions for user review (arrow, bottom left). Alternatively, the user can choose example data to load by selecting one of the links provided. Users can choose seconds, minutes or hours for runtime units. An experimental summary table displays the resulting run(s) (arrow). For each run, the number of plates (n_plates), the number of wells per plate (n_wells) and the number of measures (n_measures) are reported. Selecting one or more rows of the experiment summary table will output a line plot for each well in the MTP’s physical layout. The limits of the x and y scales are per plate.

**Figure 3:**
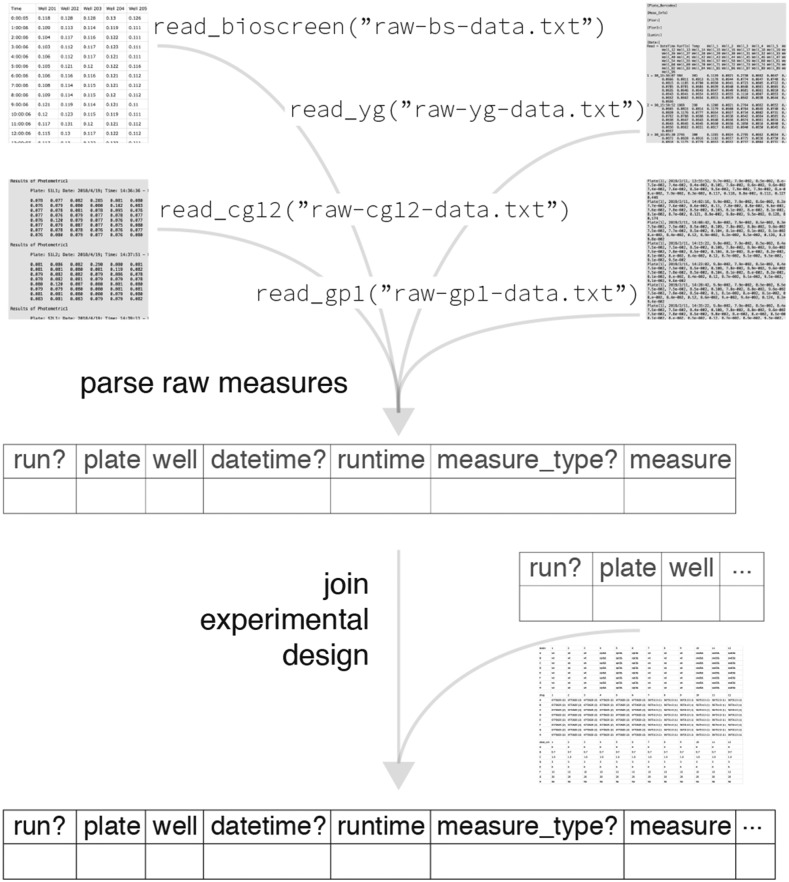
AUDIT reading modules transform varied raw input to consistent output. Examples of plain-text rendering of Bioscreen (top left), YG (top right), CG-12 (middle left) and GP1 (middle right) raw input files parsed to common tabular form. The diagram highlights optional fields with a question mark suffix. Following parsing of raw measures AUDIT joins experimental design data to the parsed measures, if available. Design files may be in generic CSV files or in plater format.

Uploaded files are automatically parsed and previewed in AUDIT (Figure 2). Measures files contain repeated measures (growth curve) data. Design files describe the distribution of experimental variables across the wells of MTPs in one or more runs. AUDIT consistently tidies varied input data to the same tabular format from one or more files (Figure 3). AUDIT records each measure in its own row with columns for the run, plate, well, runtime and measure (the value of the measurement, unit varies). Each measures file is automatically joined to one or more matching experimental design files, if provided. Design files contain the value of experiment variables for each run, plate and well. For details on the accepted formats, their specifications and joining rules see Reading and tidying raw input in Methods. The automatic parsing and merging of measures data and experimental design files makes for easier downstream analysis, distribution and archiving. This maximizes the chance that said data can be usefully re-analyzed to improve upon the findings and/or methods in the future.

#### Preprocess: Live preview provides immediate feedback on flexible user options:

**Figure 4:**
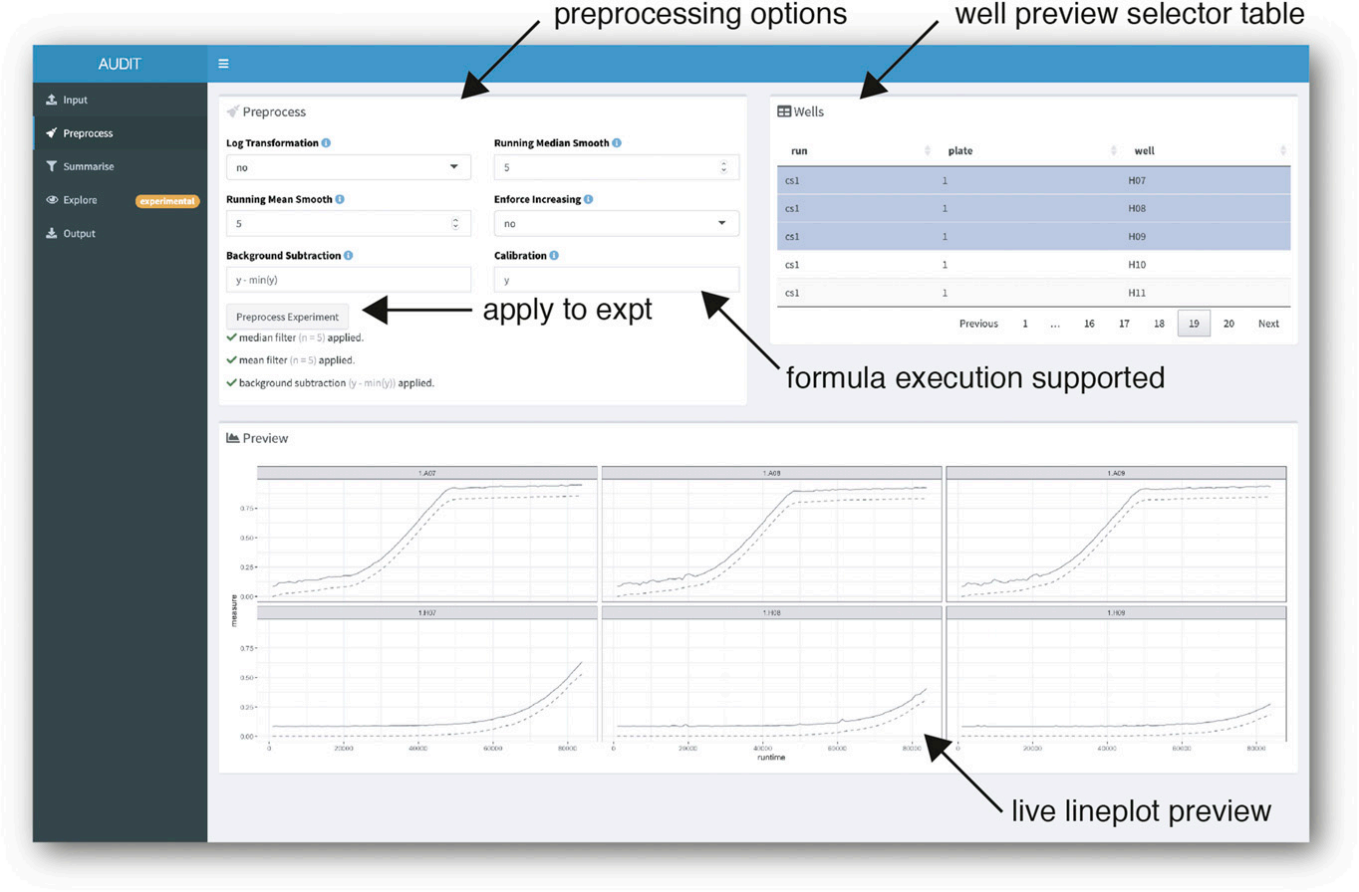
Live preview and flexible specification of varied preprocessing methods. Screenshot of preprocess panel. AUDIT makes six independent preprocessing options available (arrow, top-left). The Background Subtraction and Calibration input fields support arbitrary formula (code) execution (arrow, middle-right). The well preview selector table (arrow, top-right) contains data from selected runs in the experiment. When clicked, the ‘Preprocess Experiment’ button applies the preprocessing to the whole experiment (arrow, middle-left). To display a live plot preview, users select rows in the table. Then, AUDIT plots each well’s raw (solid line) and preprocessed measures (dashed line) (arrow, bottom-right).

AUDIT provides a live line plot preview to compare raw and preprocessed measures for any number of wells selected in the experiment (Figure 4). The user specificies the preprocessing routine with interactive selectors. First, the base for log ratio transformation, if applied. Second, the size of the windows when performing running median and mean smoothing. Next, if the data should be transformed to always be increasing. Last, background subtraction and calibration are possible with the formula execution interface provided. AUDIT binds incoming measures data for each well to the variable y. Specifying y−min(y) therefore translates each growth curve to start at y=0 by subtracting the minimum value. Other background correction and calibration methods can be specified, for more details and examples see Background correction and calibration in Methods. Live preview allows the researcher to rapidly iterate and compare preprocessing options before applying them to the entire experiment.

#### Summarize: Fit a smooth function to measures and estimate growth curve metrics with a live preview:

**Figure 5:**
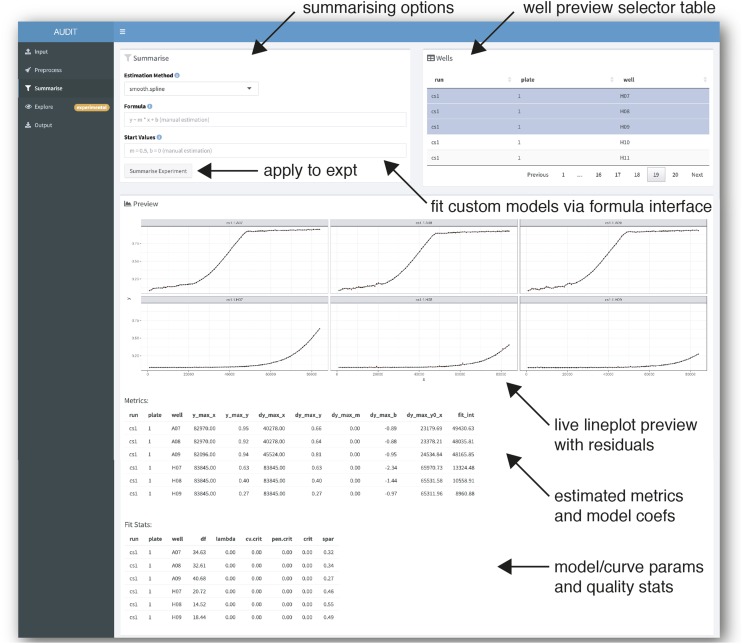
Summarize growth curves by curve fit and group wells into reference and target sets. Screenshot of summarize panel in manual mode. AUDIT enables interactive specification of arbitrary growth models. Users specify a model via R’s formula interface. AUDIT estimates model coefficients for each curve, using non-linear least-squares optimization, from user-supplied initial estimates. When clicked, the ‘Summarize Experiment’ button applies the preprocessing to the whole experiment (arrow, middle-left). The well preview selector table (arrow, top-right) contains data from selected runs in the experiment. Selecting rows in the table, will cause a live line plot preview of each curve fit (solid line) atop the underlying measures data (points). Fit residual error is also plotted (red area segment). The application displays a tabular preview of the estimated summary metrics, including model specific coefficients, and the curve quality metrics.

AUDIT enables arbitrary model fitting to preprocessed growth curves with an interactive live preview (Figure 5). It provides five estimation methods, including a ‘model-free’ cubic spline (default) and a ‘manual’ mode which enables the user to specify any growth model expressible with R’s built in formula method. From the fitted data, AUDIT computes summary metrics and estimates each model coefficient. For details on the extensible model interface and the summary metrics computed see Smooth spline interpolation and self-starting model fits in Methods. By examining a live preview of the fitted curve and the residual error, users can interactively examine the performance of different fitting procedures. As an example, we fit a Brody growth model (Brody 1966) (originally used to model economic expansion) to the example data (Figure S6).

#### Explore: Inspect the distribution of summary metrics across an experiment and within groups:

**Figure 6:**
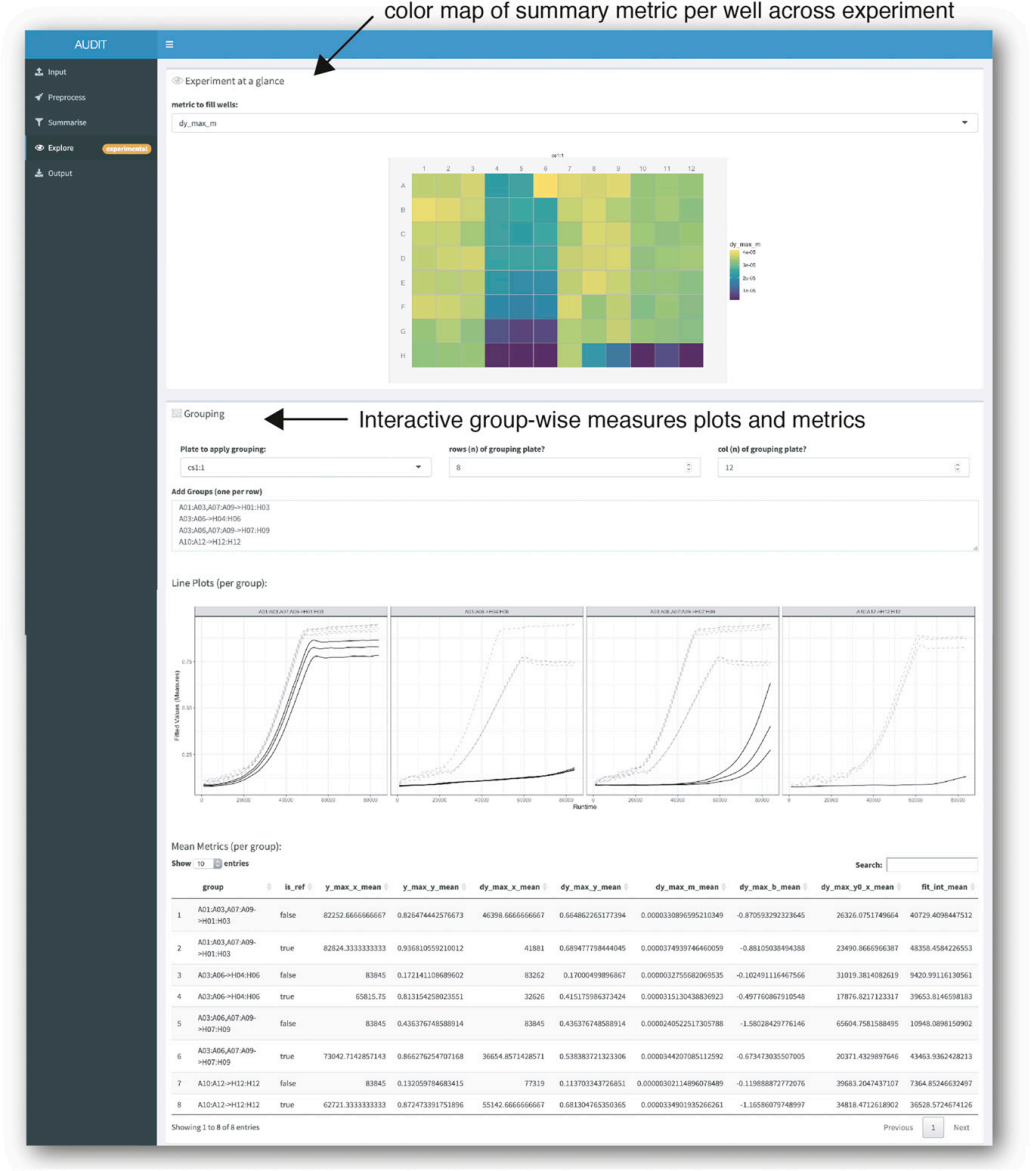
Explore the distribution of summary metrics across an experiment and by group. Screenshot of AUDIT’s explore panel. The researcher chooses a summary metric and AUDIT creates a color map across the wells in an MTP view plot (arrow, top). Users can specify Reference-target groups interactively. AUDIT will generate line plots of the fitted growth curves along with a summary table of per-group means for each numeric summary metric computed.

Estimated metrics can be visualized across an entire experiment with mtpview plots. Figure 6 shows an annotated screenshot of the explore panel. mtpview powers the experiment-at-a-glance functionality, which scales to hundreds of plates. Figure S7 shows a 144 plate experiment at a glance. This summary view provides a qualitative comparison of wells, plates and runs.

To enable within-plate comparisons and group wells in reference and target groups, AUDIT also provides an interactive shorthand. Users specify groups with ‘A01’ style notation in the following syntax: [plate name]![ref wells]->[target wells]. AUDIT automatically generates line plots of fitted measures for each group. The application also computes a table of per-group means for all numeric summary metrics. See Table S5 for examples and Applying reference/target groups in Methods for details.

#### Output: Download tidied and preprocessed measures and summary data as CSV files:

**Figure 7:**
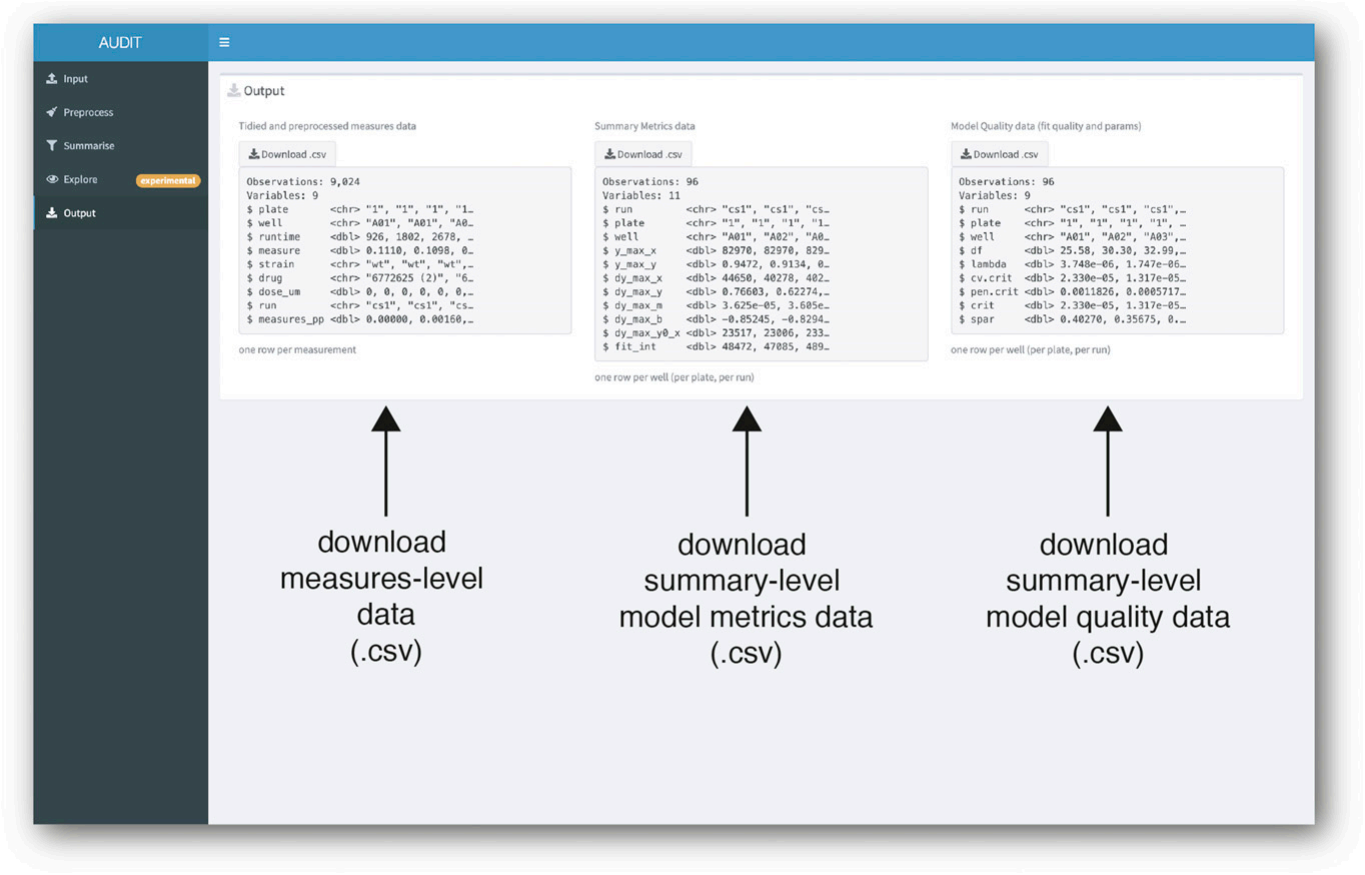
Download tidied and preprocessed measures and summary data as CSV files. Screenshot of AUDIT’s output panel. Three data files are available for download: Measures data, containing tidied raw and preprocessed measures (left), summary-level data containing estimated metrics and model components/coefficients (middle), and summary-level fit quality data (right).

Measures, summary metrics, and quality statistics are available for download from AUDIT’s output panel (Figure 7). We chose this CSV format output for easy distribution and interoperability. These data are amenable to further analysis, for example filtering based on quality of fit, in any spreadsheet-type application.

### Anticipated Results

To demonstrate AUDIT, we sourced a raw data file from our internal archive. We chose the first raw measures data file sampled for which we had information on the experimental design. In this experiment, we probed three Saccharomyces cerevisiae with each of two chemical compounds in triplicate. BY4743 and matched deletion strains opi3Δ and swd3Δ were treated with compounds identified as 5673113 (1) and 6772625 (2). An eight step two fold dilution covered a final concentration range from 50 to 0 micro Molar (uM). A GENios spectrophotometer (Tecan, Switzerland) measured Optical density at 595 nanoMeters ($OD_{595nm}$) every 15 min for 94 cycles.

Prior to the AUDIT session, we copied this raw measures file and renamed it cs1.csv. We also transferred the experimental design into plater format and saved the resulting file with a _design.csv suffix. We uploaded the resulting files to AUDIT and processed them as demonstrated in Movie S1 and in the annotated screenshots of each AUDIT panel: input (Figure 2), preprocess (Figure 4), summarize (Figure 5) and output (Figure 7). We renamed the downloaded output files with the prefix cs1_output-.

**Figure 8:**
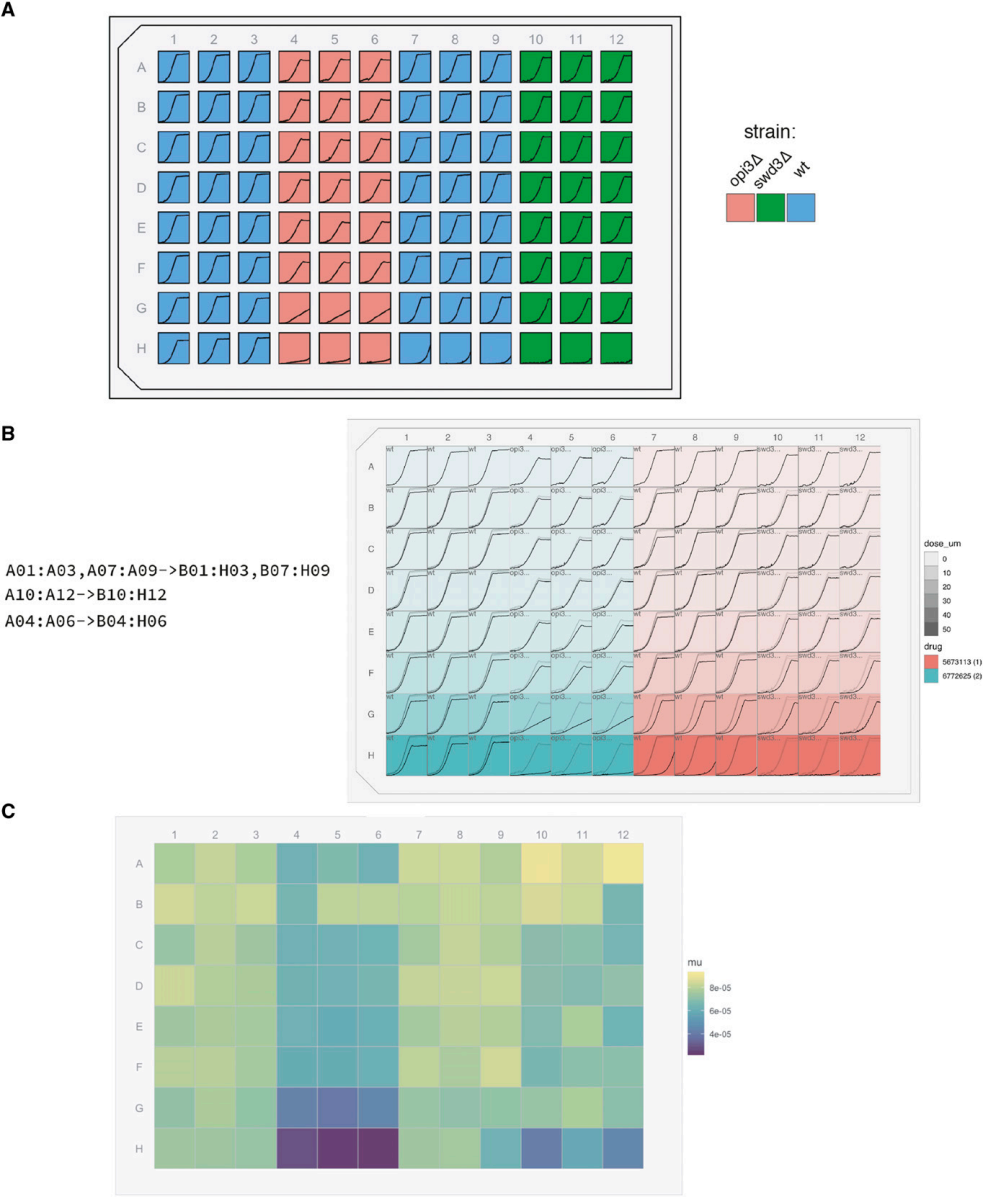
Case Study Overview. A. MTP view plot maps the values of the strain variable provided in the experimental design to the well fill color. A line plot of the measures data are plotted for each well. B. MTP view plot maps the drug identifier to the fill color and the dose in micromolar to the opacity of the rectangular wells. Line plots are according to the group specifications provided to the left of the plot. For each group, each reference curve is plotted in light, semi-transparent gray, in the target well while each the data for each target well is plotted in black. C. MTP view plot maps the values of the maximum growth rate reported in the summary metrics data. Key is provided on the right for each figure panel.

Further analysis is possible with additional functions in AUDIT’s individual modules. This functionality is not available interactively. It requires the user to call them in R, for example with the additional MTP view plots. First, visualizing the experimental design in place can speed the qualitative inspection (auditing) of growth curves (Figure 8A,B). Second, overlaying duplicate growth curves from reference wells onto target wells, using opacity to differentiate reference from target curves (Figure 8B). We provide the code required to produce these plots in Listing S5 and Listing S6, respectively.

## Discussion

Growth curve analysis can be a fast and cost-effective means of HTS, particularly when dealing with a large number of chemical probes. That said, this technique requires manipulating and inspecting data from hundreds of MTPs across dozens of runs in parallel. We present AUDIT (and the suite of R packages on which it is built) to make this possible across multiple instrument types.

Abstracting an entire growth curve to a single value has advantages and limitations. In High Throughput (HT) experiments where it becomes impractical to inspect growth curves individually, metrics provide a compressed representation. Although these summary statistics can ease comparison and ranking across thousands of conditions, they are also lossy. They do not necessarily capture the underlying biology. For example, if the preprocessed value used to measure growth (*e.g.*, OD) does not perfectly relate to cellular concentration (or density), the metrics reported (*e.g.*, maximum growth rate) do not represent the population growth rates. Therefore, researchers should carefully preprocess their data for metrics to directly map to the biological system being studied. Nonetheless, a quantitative approach that summarizes growth curves to one or more metrics can be useful in HTS applications.

We designed AUDIT to be extended. We therefore mark two areas for near term development. First, writing parsing modules for other data formats not currently part of AUDIT. Parsed and tidied data from custom or commercial instruments should adhere to AUDIT’s conventions for storing repeated measures experiment data. For this purpose, the output format should be plain-text human- and computer-parsable where possible. Second, writing new modules to help the researcher perform quality control within AUDIT. For example, integrating heuristics that display or check instrument-specific parameters, such as temperature control and spatial effects. We already include a basic temperature check module in the explore panel.

We designed AUDIT’s underlying modules to be remixed. We highlight new directions for potential future developments. It is possible that better tracking of experimental design variables could enable automated modeling of the ‘history of the inoculum’ and perhaps minimize spurious repetitions. For example, If an experiment calls for a previously characterized inoculum, it could be omitted. Next, the integration of historical databases or archives could enable automated outlier detection and interactive browsing by collaborators or non-specialized researchers. Specifying the experimental design ahead of time and performing Just-in-time analysis during the run could replace post-hoc analysis and provide a valuable in-run diagnostic. This could enable direct instrument feedback. AUDIT could shorten, lengthen or repeat a run to meet a predefined clearance between the reference and target wells of the specified groups.
